# Cell-wall deficient *L. monocytogenes* L-forms feature abrogated pathogenicity

**DOI:** 10.3389/fcimb.2014.00060

**Published:** 2014-05-20

**Authors:** Barbara Schnell, Titu Staubli, Nicola L. Harris, Gerhard Rogler, Manfred Kopf, Martin J. Loessner, Markus Schuppler

**Affiliations:** ^1^Department of Health Sciences and Technology, Institute of Food, Nutrition and Health, Swiss Federal Institute of Technology Zurich (ETHZ)Zurich, Switzerland; ^2^School of Life Sciences, The Global Health Institute, Swiss Federal Institute of Technology Lausanne (EPFL)Lausanne, Switzerland; ^3^Division of Gastroenterology and Hepatology, University Hospital ZurichZurich, Switzerland; ^4^Department of Biology, Institute of Molecular Health Sciences, Swiss Federal Institute of Technology Zurich (ETHZ)Zurich, Switzerland

**Keywords:** L-forms, *L. monocytogenes*, innate immunity, pathogenicity, virulence genes

## Abstract

Stable L-forms are cell wall-deficient bacteria which are able to multiply and propagate indefinitely, despite the absence of a rigid peptidoglycan cell wall. We investigated whether L-forms of the intracellular pathogen *L. monocytogenes* possibly retain pathogenicity, and if they could trigger an innate immune response. While phagocytosis of *L. monocytogenes* L-forms by non-activated macrophages sometimes resulted in an unexpected persistence of the bacteria in the phagocytes, they were effectively eliminated by IFN-γ preactivated or bone marrow-derived macrophages (BMM). These findings were in line with the observed down-regulation of virulence factors in the cell-wall deficient *L. monocytogenes*. Absence of Interferon-β (IFN-β) triggering indicated inability of L-forms to escape from the phagosome into the cytosol. Moreover, abrogated cytokine response in MyD88-deficient dendritic cells (DC) challenged with *L. monocytogenes* L-forms suggested an exclusive TLR-dependent host response. Taken together, our data demonstrate a strong attenuation of *Listeria monocytogenes* L-form pathogenicity, due to diminished expression of virulence factors and innate immunity recognition, eventually resulting in elimination of L-form bacteria from phagocytes.

## Introduction

*Listeria monocytogenes* is a Gram-positive pathogen ubiquitously found in natural environments. The bacteria are able to switch from a saprophytic to an intracellular lifestyle, thereby causing Listeriosis, a food-borne disease with a fatality rate of up to 30%, affecting young, old, pregnant, and immunocompromised individuals (Sleator et al., 2009). The hallmarks of *Listeria* pathogenicity and virulence have been intensively studied, and are reviewed and summarized elsewhere (Cossart, 2011).

Pathogen recognition and induction of immune responses are important prerequisites in fighting bacterial infections. The innate immune system can sense pathogen-associated molecular patterns (PAMPs), by evolutionary conserved host sensors known as pathogen recognition receptors (PRRs) (Kumar et al., 2011). However, pathogens such as *Listeria monocytogenes* have developed strategies to evade or modulate these immune defenses in order to ensure their survival (Corr and O'Neill, 2009). The innate immune recognition of *L. monocytogenes* at the cell surface or inside phagosomes of macrophages and dendritic cells (DC) is mediated by Toll-like receptors (TLR-2, 4, 5, and 9), whereas recognition within the cytosol following their escape from the phagosome is conferred by nucleotide-binding oligo-dimerization domain (NOD)-like receptors (NOD-1, NOD-2, and NLRP 3-4) (Eitel et al., 2011). Consequently, a distinct cytokine response can discriminate vacuolar and cytosolic localized *L. monocytogenes* (Leber et al., 2008). Vacuole-entrapped *L. monocytogenes* activate a MyD88-dependent induction of pro-inflammatory cytokines (IL-1α, IL-1β, TNF-α, and other cytokines/chemokines), whilst the cytosolic recognition is characterized by an IFR-3/TKB1-dependent induction of Type-I interferons (Stockinger et al., 2004; Leber et al., 2008). Accordingly, Interferon-β (IFN-β) is thought to be exclusively induced upon cytosolic entry of pathogenic *L. monocytogenes*, and not by avirulent mutants that lack the ability to enter the cytosol (O'Riordan et al., 2002; Stockinger et al., 2002).

L-forms are cell wall-deficient variants of bacteria, which have lost the ability to maintain or rebuild a rigid peptidoglycan envelope, as a result of exposure to cell-wall active antibiotics. It was shown that L-forms of *Listeria monocytogenes* and other bacteria are not only able to survive, but switch to an alternative life-style to replicate and grow as stable cell lines without the presence of antibiotics (Dell'Era et al., 2009; Briers et al., 2012). Since peptidoglycan serves as an important molecular pattern for the innate immune system to recognize the presence of bacteria, the question arises as to whether the lack of a mature cell wall disables the host to induce an appropriate immune response against L-forms. Unfortunately, next to nothing is known about the expression of virulence genes in L-form bacteria, or the recognition of the wall-less cells by the immune system. This is intriguing, in particular in regard to numerous studies on experimental infections claiming a possible role for L-forms in diseases such as endocarditis, rheumatic fever, Crohn's disease, sarcoidosis, septicemia, urinary tract infections, or chronic gastritis (Owens, 1987; Markova et al., 1997; Michailova et al., 2000, 2007). However, direct evidence for a role of L-forms in disease is still missing, as re-isolation from an infected host has not yet been reported (Onwuamaegbu et al., 2005). Generally, investigation of the pathogenic properties of L-forms originating from pathogens is needed to determine their potential role in human disease.

The aim of this study was to investigate the pathogenic traits of *L. monocytogenes* L-forms. Interaction of L-forms with macrophages was assessed by the use of confocal laser-scanning microscopy (CLSM), and the innate immune response was monitored by measuring pro-inflammatory cytokines, using ELISA and Real-Time quantitative Reverse Transcription PCR (Real-Time qRT-PCR). Our findings indicate a severe attenuation of virulence of bacteria in the L-form state.

## Materials and methods

### Bacteria

All *Listeria* strains (Table 1) were grown in brain-heart infusion medium (BHI; Biolife, Italy), unless indicated otherwise. Induction of L-forms was performed according to a previously published protocol, with slight modifications (Dell'Era et al., 2009). Briefly, overnight cultures of parental *Listeria* strains were diluted 1:50 in fresh medium and further incubated for 2 h at 30°C. Aliquots of 50–100 μ l were then inoculated into *Listeria* L-form soft agar medium (LLM) [3.7% BHI (Biolife, Italy), 15% sucrose, 2.5% MgSO_4_ × 7 H_2_O, and 3% milk serum powder, 3% agar], supplemented with 50 μg/ml Penicillin G (Sigma Aldrich, Germany), and incubated at 32°C until small irregular L-form colonies were macroscopically visible. Cells were then serially transferred to tubes containing fresh LLM soft agar, with gradually reduced Penicillin G concentration (50, 25, 12.5, 0 μg/ml), and incubated until the L-form cells were stabilized, i.e., did not show reversion in medium without drug supplement. All further experiments were performed using stable *Listeria* L-form cultures maintained in LLM without antibiotics. Enumeration of parental *Listeria* was based on OD_600_ absorbance, and verified by serial dilution plating. L-form cells were quantified based on single copy gene amplification by Real-Time qPCR (Dell'Era et al., 2009), using L-forms washed with protoplast buffer (1 M sucrose, 100 mM NaCl, 50 mM Tris-HCl, 10 mM Mg_2_Cl_2_), and centrifuged for 2 min at 1000 g.

**Table 1:** **Plasmids and strains used in this study**.

**Strains and Plasmids**	**Description**	**Sources or References**
**PLASMIDS**		
pAUL-A	Vector plasmid for site-directed mutation	Chakraborty et al., 1992
pAD-cCFP	pPL2-Phyper-CFP (constitutive)	Balestrino et al., 2010
pPL2(P_HYP_)-GFP	pPL2-Phyper-GFP (constitutive)	Balestrino et al., 2010
***L. monocytogenes* strains**		
EGDe	Wild type, serovar 1/2a, ATCC BAA-679	Glaser, 2001
EGDe::pPL2(P_HYP_)-GFP	pPL2(P_HYP_)-GFP chromosomally integrated	J. Kreft, this study^*^
EGDe Δ*actA*::pPL2(P_HYP_)-	Deletion of *actA*, constitutive GFP expression	J. Kreft, this study^*^
EGDe Δ*inlA/B*::pPL2(P_HYP_)-	Deletion of *inlA/B*, constitutive GFP expression	J. Kreft, this study^*^
EGDe Δ*prfA*::pPL2(P_HYP_)-	Deletion of *prfA*, constitutive GFP expression	J. Kreft, this study^*^
EGDe Δ*iap*::pPL2(P_HYP_)-GFP	Deletion of *iap*, constitutive GFP expression	J. Kreft, this study^*^
ScottA	Wild type, serovar 4b	Fleming et al., 1985
ScottA::pPL3-GFP	pPL3-GFP chromosomally integrated	Dell'Era et al., 2009
ScottA Δ*hly*	In-frame deletion of *hly*	This study
ScottA Δ*hly*::pPL3-GFP	pPL3-GFP chromosomally integrated	This study
WSLC 1042	Wild type, serovar 4b	Weihenstephan *Listeria*
WSLC 1042::pAD-cCFP	pPL2(P_HYP_)-CFP chromosomally integrated	This study
***L. innocua* strains**		
WSLC 2257	Wild type, serovar 6a	Weihenstephan *Listeria*
WSLC 2257::pPL2(P_HYP_)-	pPL2(P_HYP_)-GFP chromosomally integrated	This study
***Escherichia coli***		
XLl-Blue MR'	Used for plasmid manipulations	Stratagene, USA

* *Knockout mutant strains provided by J. Kreft, cloning of pPL2(P_HYP_)-GFP was performed in this study*.

### Cells

Murine P-388D1 macrophages (DSMZ, Koren et al., 1975) and human THP-1 cells (DSMZ, Auwerx, 1991) were grown in RPMI-1640 (Sigma Aldrich, Germany) + 10% FBS (PAA, Austria) in a humid chamber at 37°C and 10% CO_2_ (Thermo Electron Corporation, Hepa 100 filter). THP-1 cells were differentiated using 5 μg/ml PMA (Sigma Aldrich, Germany) 2 h prior to infection.

Bone marrow-derived macrophages (BMM) were isolated from C57BL/6 mice (University Hospital Zurich, Switzerland). DC were isolated from C57BL/6 mice and respective MyD88-deficient/mutated mice (ETH Zurich, Switzerland). Isolation, differentiation and culture was performed as described previously (Shin et al., 2008). In brief, freshly prepared mice hip and leg bones were capped, and bone marrow cells carefully flushed out and stored in Dulbecco's modified Eagle medium (DMEM; Sigma Aldrich, Germany) containing 10% FBS (PAA Laboratories, Austria) and 1% Pen/Strep (Sigma Aldrich, Germany). Collected cells were centrifuged (10 min 1000 g at 4°C) and resuspended in 20 ml medium prior to filtration through a 40 μm cell strainer (BD bioscience, USA). BMM were counted and diluted to 10^6^ cells per well. For differentiation, 20 ng/ml GM-CSF (Biovision, USA) was added at day 0, 4, and 7 post inoculations. Confirmation of the grade of differentiation and determination of the time point when the cells could be used for further experiments was done by morphological analysis. Prior to use in challenge experiments with bacteria, antibiotics were removed by washing three times with fresh DMEM. Animal experimental procedures were approved by the local animal ethics committee (Zurich).

### Genetic manipulations

Construction of cytosolic GFP-expressing *Listeria* was performed by single copy insertion of plasmid pPL2(P_HYP_)-GFP into a tRNA-Arg locus mediated by bacteriophage PSA site-specific integrase. The vector remains stable in the absence of drug selection, and does not cause polar effects (Lauer et al., 2002).

To create a *hly* knockout mutant, the temperature-sensitive shuttle vector pAUL-A (Chakraborty et al., 1992) was employed. Site-directed mutagenesis was performed by splice-overlap extension PCR (Hornton et al., 1990). Primers are detailed in Table S1 of Supplementary Material. Resulting DNA fragments of approx. 1000 bp were digested using *Sac*I and *Bam*HI, ligated into pAUL-A, and cloned in *E. coli* XL1-Blue (Stratagene, USA). The plasmid was transferred to *Listeria* to create a knockout in *hly* following homologous recombination with the bacterial chromosome as described (Chakraborty et al., 1992). Candidate clones were screened by PCR using primers hly-x, hly-y and hly-z (Table S1 of Supplementary Material). Presence of a 450 bp fragment generated from primers hly-x and hly-z, together with absence of a fragment using primers hly-x and hly-y, indicated successful mutagenesis and deletion of the *hly* target region.

### Monitoring intracellular L-form cells

Murine P-388D1 macrophages (DSMZ ACC 288, DSMZ, Germany) and human THP-1 macrophages (DSMZ ACC 16) were cultured in multi-well glass-bottom chambers (Ibidi μ-chambers, Vitaris, Switzerland), in 90% RPMI 1640 + 10% FBS, and incubated at 37°C in a 5% CO_2_ gas atmosphere. P-388D1 macrophages (3 × 10^6^ cells/sample) were challenged with GFP-producing parental (MOI: 50) and L-form (MOI: 1000) *L. monocytogenes* ScottA::pPL3-GFP, previously washed twice in cell culture medium. Following incubation for 2 h at 37°C, cultures were washed five times using cell culture medium, prior to addition of gentamicin (50 μg/ml; Sigma Aldrich, Germany), for 1 h. After replacing the gentamicin with fresh medium, cultures were screened (10-fold magnification) for GFP-expressing L-forms inside phagocytes, using of a confocal laser scanning microscope (Leica TCS-SPE, Leica Microsystems, Germany), equipped with an incubation chamber set to 37°C. Quantification of intracellular L-forms was performed by enumeration of GFP signals in five randomly selected optical fields for each time point. Viability and metabolic activity of intracellular L-forms was assessed by addition of 2 μg/ml CTC (5-cyano-2,3-ditolyl tetrazolium chloride; Fluka, Germany) for 1 h, and washing twice with medium prior to microscopy.

### Detection of GFP-expressing L-forms in bone marrow-derived macrophages

BMM were cultured in 90% DMEM medium (Sigma Aldrich, Germany) including 10% FBS (PAA Laboratories, Austria) and 1% Pen/Strep (Sigma Aldrich, Germany), prior to challenge with GFP-expressing L-forms and gentamicin treatment as described above. L-forms were suspended in DMEM containing 50 μg/ml Penicillin G (Sigma Aldrich, Germany) and 10 μg/ml Chloramphenicol (Sigma Aldrich, Germany), and added to the BMM, which had been washed previously three times with PBS. Because of the strong autofluorescence of BMM in the emission spectrum typical for GFP, detection of GFP-expressing L-forms had to be performed by an immunofluorescence assay using an anti-GFP Alexa555 antibody. For this purpose, 24 h post infection of BMM an anti-GFP Alexa555 antibody (Invitrogen, USA) was applied, followed by observation by CLS microscopy.

### Spatial localization of intracellular L-forms

In order to determine whether L-forms detected in macrophages were located within primary vacuoles or phagolysosomes and to trace phagolysosomal fusion LysoTracker Yellow HCK-123, a yellow fluorescent dye which stains acidic compartments in live cells with excitation/emission maxima ~465/535 nm (Invitrogen, USA), was applied. Because of partial overlap in the emission spectrum of GFP and LysoTracker Yellow, a *L. monocytogenes* strain 1042 L-form with constitutive expression of cyan fluorescent protein (CFP) from plasmid pAD-cCFP (Balestrino et al., 2010) was created and used in combination with LysoTracker. L-forms (MOI: 1000) or heat-inactivated parental bacteria (MOI: 50) were exposed to P-388D1 macrophages (3 × 10^6^ cells/sample). After different time points, the macrophage cultures were stained with 50 μM LysoTracker Yellow for 45 min at 37°C, washed twice in medium and analyzed by CLSM.

To exclude that macrophages containing viable L-forms were apoptotic and therefore not able to eliminate persisting L-forms, an *in situ* Cell Death Detection Kit (Roche, Switzerland) was employed, according to the recommendations of the manufacturer. Early apoptotic stages are characterized by the occurrence of single- and double-stranded DNA breaks, which can bind fluorescein-dUTP at free 3′-OH groups. After staining and washing, samples were analyzed by confocal laser scanning microscope for apoptotic cells that incorporated the fluorescent label.

### Virulence gene expression in *Listeria* L-forms

P-388D1 macrophages (3 × 10^6^ cells/sample) were challenged for 3 h at 37°C with fresh parental (MOI: 50) and L-form (MOI: 1000) *L. monocytogenes* ScottA::pPL3-GFP prior to gentamicin treatment and harvesting the cells in 1 ml Trizol™ (Life Technologies, Switzerland) for later RNA extraction (Hedge et al., 2000). DNA-free RNA was obtained using RNeasy™ Mini Kit and RNase-free DNase (Qiagen, Germany). Complementary DNA (cDNA) was generated from purified RNA using TaqMan Reverse Transcription Reagents (Applied Biosystems, USA). Quantitative RT-PCR was performed with 400 ng/reaction of primers (listed in Table S1 of Supplementary Material), 5 μl 2 × SYBR™ Green MasterMix (Applied Biosystems, USA), 1 μl water and 2 μl of sample, in a Rotor Gene 6000 instrument (Corbett Robotics, USA). PCR was at the following parameters: 1 × 95°C for 10 min; 50 × (10 s at 95°C; 15 s at 58°C; 20 s at 72°C), with a single fluorescence measurement, and melting curve analysis from 50 to 95°C at 1°C s^−1^. Expression of target genes (*actA*, *dacA*, *hly*, *hpt*, *iap*, *lgt*, *pgdA*) was calculated based on the number of *L. monocytogenes* genome copies, and expressed as a ratio to the *gyrA* single copy control gene.

### Determination of ActA on L-form cells

The membrane-anchored ActA protein was extracted from bacterial cells according to a previously published protocol (Kocks et al., 1992), with modifications. Parental *L. monocytogenes* ScottA::pPL3-GFP were grown overnight at 37°C to stationary phase, in BHI supplemented with 0.2% activated charcoal (Fluka, Germany). Aliquots of 200 ml were centrifuged (4°C, 500 g for 20 min), and cells washed three times with 100 ml PBS. The pellet was resuspended in 3 ml PBS containing 1% SDS (Biochemika, Germany), and incubated with constant rotation (15 min at RT) until cell lysis occurred. L-form *L. monocytogenes* ScottA::pPL3-GFP were grown in LLM soft agar (4 ml) containing 0.2% activated charcoal (Fluka, Germany) overnight at 37°C for 10 days. Then, the content of ten entire LLM soft agar tubes was pooled, washed twice with 100 ml protoplast buffer at 4°C (500 g for 20 min), resuspended in 3 ml PBS containing 1% SDS (Biochemika, Germany), and incubated on a rotator for 5 min at RT. Both preparations were then centrifuged at 15,000 g for 10 min at 4°C. The protein supernatant was concentrated (Vivaspin 500, 50 kDa cut off; Sartorius, Switzerland), and standardized (BCA™ protein assay kit, Thermo Scientific, USA). Further concentration of ActA protein was performed overnight at 4°C, using 150 mg/sample magnetic Protein-A-labeled paramagnetic nano-sized beads (TurboBeads; Zurich, Switzerland), coated with *L. monocytogenes* anti-ActA antibodies (polyclonal rabbit anti-ActA serum kindly provided by M. Footer, Stanford University, USA). The concentrated protein preparations were separated using PAGE on 12.5% Tris-HCL gels (Criterion; BioRad, Switzerland), for 2 h at 125 V. Proteins were then transferred by western blotting onto nitrocellulose membranes (iBlot system, Life Technologies; USA) according to the instructions of the manufacturer. The membrane was blocked for 1 h at RT in TBST containing 10% (w/v) milk powder. After three washes for 10 min at RT in TBST, polyclonal rabbit anti-ActA was added at a 1:20,000 dilution, in TBST containing 1% (w/v) milk powder and incubated for 1 h at RT. After washing, a HRP-conjugated secondary antibody (goat-anti rabbit antibody; Calbiochem, VWR, Switzerland) was added 1:20,000 in TBST containing 1% (w/v) milk powder, for another hour. Following three times washing, the substrate (Luminata™ Crescendo Western HRP substrate; Millipore Billerica, USA) was added. Chemiluminescence was recorded using a Kodak Image Station IS200R (Carestream Health, USA).

### Cytokine induction in macrophages

P-388D1 macrophages were grown for 4 days (semi-confluence), in 90% RPMI 1640 + 10% FBS at 37°C and 5% CO_2_, using six well plates (SPL Life Science, Switzerland). Activated macrophages (100 ng/ml IFN-γ) were incubated with either parental or L-form *L. monocytogenes* ScottA::pPL3-GFP (MOI: 50). LPS (100 ng/ml) was used to create activation positive controls. Negative controls received soft agar or pure culture medium only. After 6 h of incubation at 37°C and 5% CO_2_, two each of the six well plates (corresponding to 3 × 10^6^ cells) were harvested. Macrophages were washed once with 2 ml PBS and pooled in 1 ml TRIzol™ (Invitrogen, USA), and frozen at −80°C. For RNA extraction, samples were thawed on ice, 200 μl chloroform added, and samples vortexed thoroughly for 1 min prior to incubation at RT for 2.5 min and centrifugation at 12,000 g and 4°C for 15 min. After resuspension of the RNA pellet in 50 μl RNase-free water, DNA-free RNA was obtained by DNase treatment and spin-column purification (RNeasy; Qiagen, Germany) according to the recommendations of the manufacturer. Quantity and purity of the RNA preparations was determined using a NanoDrop spectrophotometer (ThermoFischer Scientific, USA). cDNA was generated by reverse transcription (TaqMan Reverse Transcription Reagents; Applied Biosystems, USA). Quantitative Real-Time PCR was performed in duplicates using primers listed in Table S1 of Supplementary Material. Cytokine expression values of five replicates were determined in relation to the expression of the single copy housekeeping gene *β-actin*. Expression values determined for the controls (soft agar, medium) were subtracted from the values determined for challenged macrophages.

### Challenge of WT and MyD88-deficient dendritic cells with parental and L-form *L. monocytogenes*

DC of wild type and MyD88-deficient mice were seeded to 24 wells (5 × 10^5^ cells per well), adapted overnight and challenged with parental and L-form *L. monocytogenes* ScottA::pPL3-GFP (MOI: 50). Supernatants were harvested 24 h post infection and further processed to determine IL-6 by quantitative ELISA for analysis of cytokine levels according to the manufacturer's protocol (FlowCytomix Multiplex assay, eBioscience, USA).

### Infection of mice with *L. monocytogenes* L-forms

Parental *L. monocytogenes* ScottA were grown in LLM broth at 37°C, and cfu determined as described above. After washing three times with protoplast buffer (1 M sucrose, 100 mM NaCl, 50 mM Tris-HCl, 10 mM Mg_2_Cl_2_), parental bacteria were resuspended to 100 cfu per 0.5 ml protoplast buffer containing 1/3 (v/v) soft agar. *Listeria monocytogenes* ScottA::pPL3-GFP L-forms were grown for 7 days at 37°C in LLM soft agar containing 0.16% instead of 0.3% agar, washed three times with protoplast buffer, and adjusted to 10^8^ cfu (L-form high dose) or 10^5^ cfu (L-form low dose) per 0.5 ml buffer.

Groups of 10 of sex and age-matched 8–12 week old BALB/c mice were infected by injection of 0.5 ml protoplast buffer containing the respective bacteria via the intraperitoneal (i.p.) route. Control mice received 0.5 ml protoplast buffer containing 1/3 (v/v) soft agar alone. Mice were sacrificed after 1, 3, or 6 days, and the liver and spleen removed. Both organs were divided into two equal sections and either homogenized into Trizol for later RNA extraction and Real-Time qRT-PCR analysis of cytokines, or used for bacterial plating and estimation of organ cfu (for parental bacteria only). Blood was also collected, allowed to coagulate, and serum recovered for analysis of cytokine levels.

Animal experiments were approved by the Swiss Federal Office for Veterinary Affairs (Affaires Vétérinaires, 1066 Epalinges, Canton Vaud, Switzerland), with the authorization Number 2238 according to the guidelines set by the Service de la Consommation et des Affaires Vétérinaires Federal.

## Results

### The PrfA regulon is attenuated in *Listeria monocytogenes* L-forms

**Figure 1:**
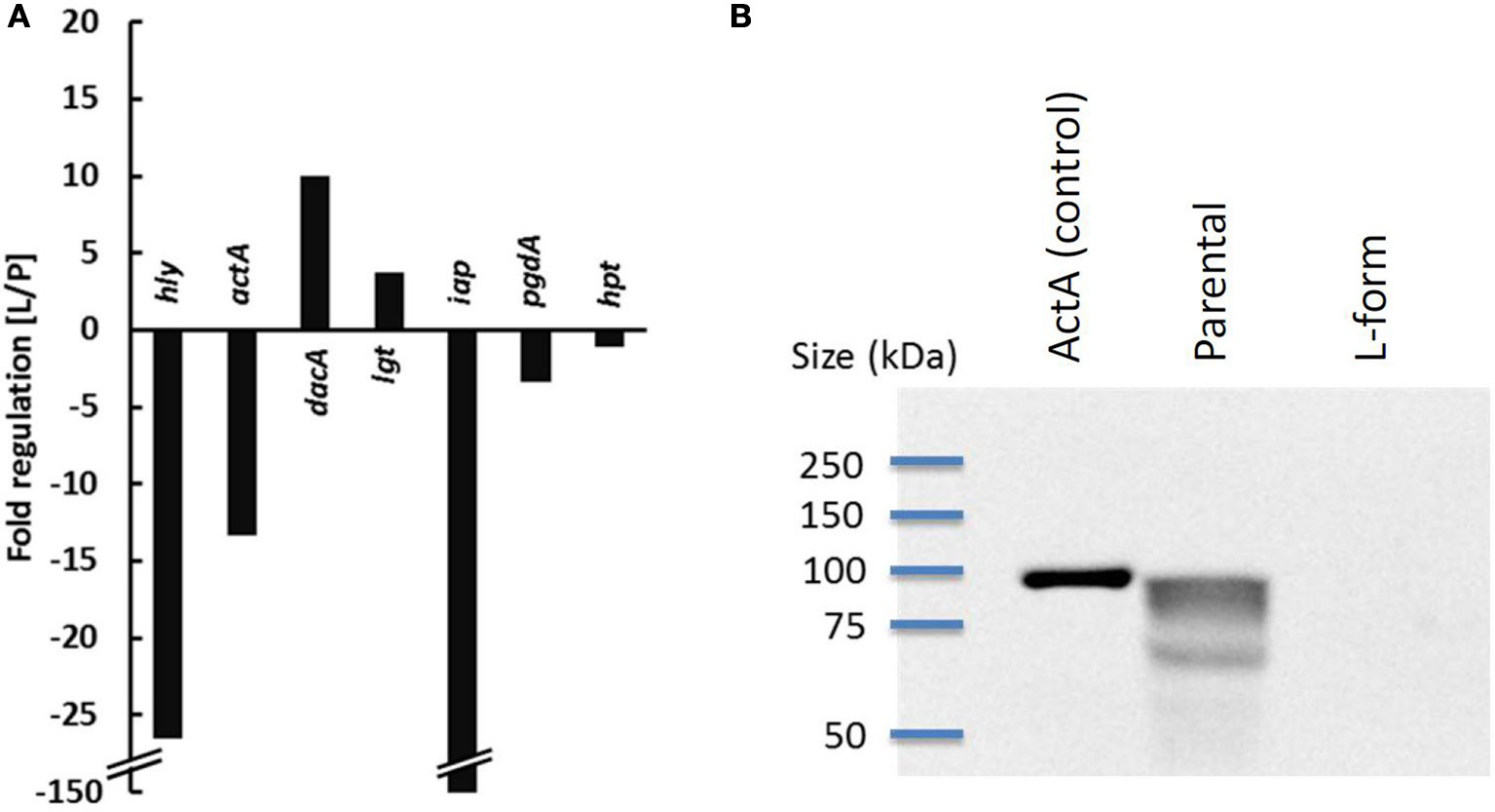
**Expression of virulence genes of parental (P) and L-form (L) *L. monocytogenes* ScottA-GFP 3 h post infection of P-388D1 macrophages determined by Real-Time qRT-PCR**. The expression of *hly, actA, dacA, lgt, iap, pgdA*, and *hpt* is depicted as x-fold up- or down-regulation in L-forms, compared to parental *Listeria* **(A)**. Detection of ActA by Western Blot **(B)** revealed its absence on L-forms.

In order to quantify and compare the expression of virulence genes in *L. monocytogenes* L-forms and parental bacteria in cell culture, Real-Time qRT-PCR analysis was performed on samples collected 3 h post infection. The results revealed a strong down-regulation of *hly* (26-fold) and actA (13-fold) which are under control of PrfA (Figure 1A). Because ActA is also proposed to be involved in autophagy recognition of bacteria (Yoshikawa et al., 2009), its presence on L-forms was further investigated by Western blot, revealing that ActA is below detectable levels on the surface of L-form cells (Figure 1B). While expression of the *iap* gene revealed strongest (150-fold) attenuation, expression of *hpt* and *pgdA* was only slightly affected, and not significantly different from parental bacteria. Higher mRNA levels were observed for *lgt* (4-fold), and *dacA* (10-fold).

### Low-frequency persistence of *Listeria monocytogenes* L-forms in macrophages

**Figure 2:**
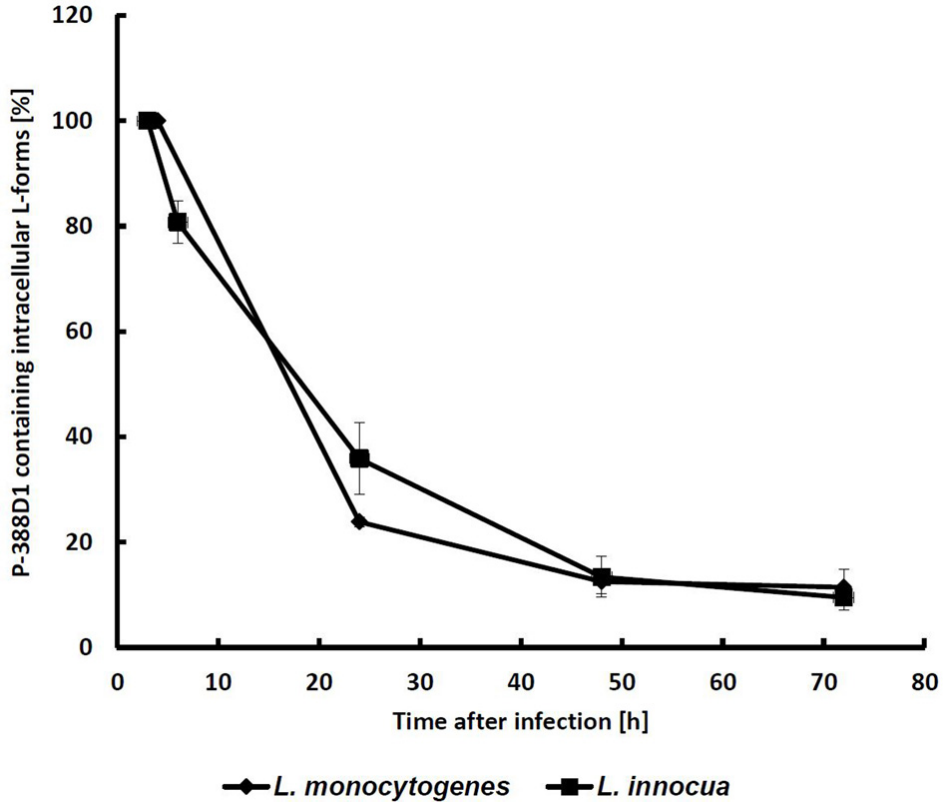
**Monitoring of GFP-labeled intracellular L-forms (ICL) in P-388D1 macrophages**. After 3 h p.i. approx. 1.2% of the macrophage population featured intracellular L-forms, which was set as the initial 100% value. Fate of the ICL was monitored for 72 h. Values represent the means of five microscopic fields in two replicate experiments. Standard deviation is indicated by error bars.

To monitor the fate of L-forms in macrophages, a gentamycin protection assay was performed using murine P-388D1 macrophages and GFP-tagged L-forms. After killing of extracellular bacteria by gentamicin, the majority of L-form cells had disappeared. However, approximately 1% of the macrophage population contained persistent intracellular L-forms (ICL). This initial number of ICL was then set as 100%, and the fate of ICLs further monitored by microscopic inspection over 3 consecutive days. During this time, the numbers of ICLs decreased to 22% after 24 h, and to 11% after 72 h (Figure 2). Interestingly, no difference was found for L-forms of *L. monocytogenes* and those from apathogenic *L. innocua*.

**Figure 3:**
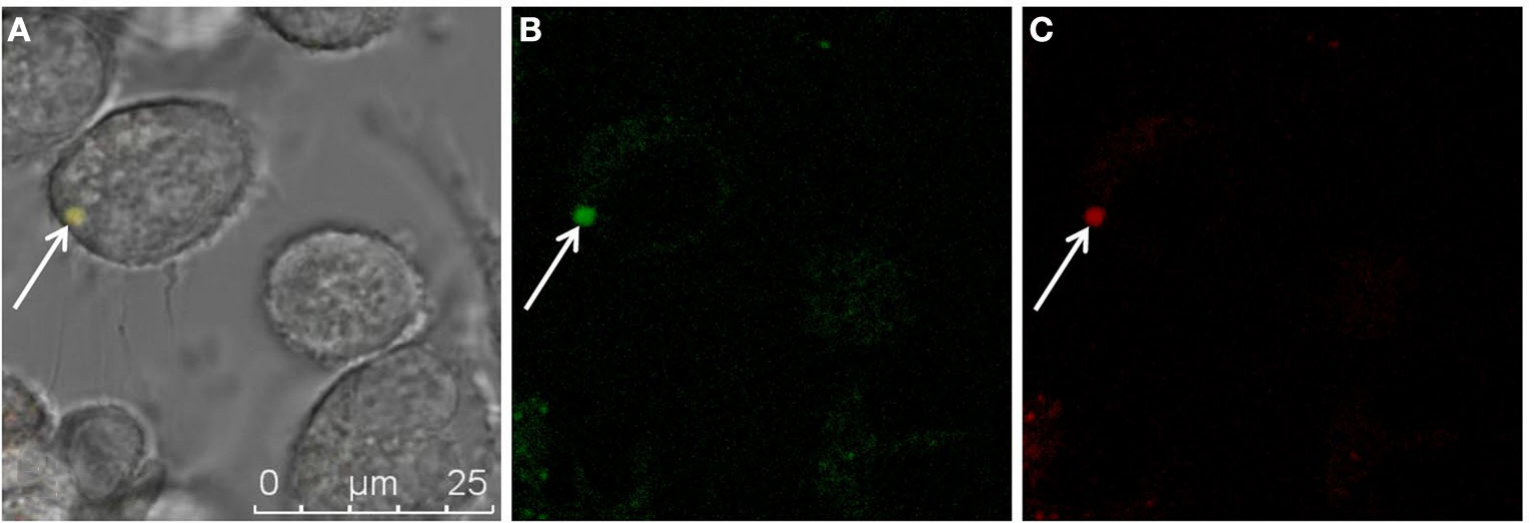
**Superimposition of fluorescent micrographs (A) showing co-localization of green fluorescence (B) from a *L. monocytogenes* ScottA-GFP cell (white arrow), and red fluorescence (C) from CTC as a marker for actively respiring bacteria within a P-388D1 macrophage after gentamicin treatment and 72 h incubation**. Scale bar applies to all images.

**Figure 4:**
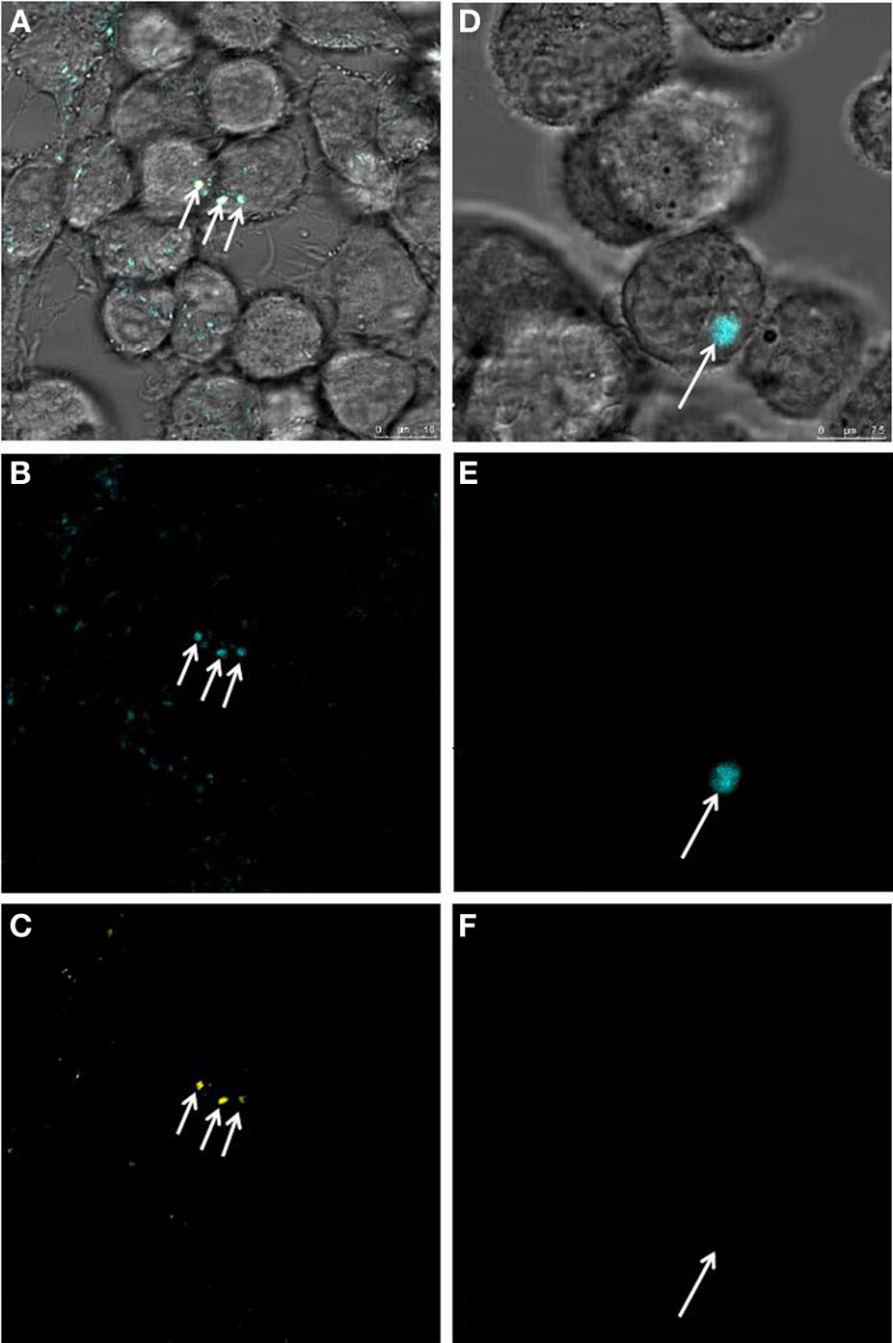
**Investigation of phagolysosomes (yellow) stained with a pH-sensitive dye for presence of *L. monocytogenes*-CFP parental, or L-form cells (blue)**. Superimposition of micrographs **(A)** shows co- localization of blue signals from heat inactivated parental *L. monocy- togenes*-CFP **(B)** with yellow signals **(C)** pointing to phagolysosomes harboring parental bacteria (white arrows). Superimposition of images **(D)** demonstrates presence of a blue signal from a *L. monocytogenes* L-form **(E)** and absence of a yellow signal **(F)**, suggesting that the intracellular *L. monocytogenes*-CFP L-form cell (white arrow) is not contained within a phagolysosome. Scale bars apply to the respective column of images.

Because GFP is not suitable as a viability marker since the protein remains detectable even in dead cells (Tsien, 1998), the redox dye CTC was employed as a viability stain (Bartosch et al., 2003) for microscopic detection of metabolizing L-forms. Co-localization of red (CTC) and green (GFP) signals strongly suggests viability of the ICL (Figure 3). Further staining was performed to investigate whether CFP-expressing ICL were contained by phagolysosomes (Figure 4). In contrast to parental bacteria (Figures 4A–C), CFP-signals from L-forms (Figure 4E) did not co-localize with phagolysosome signals (Figure 4F), suggesting that ICL were not inside phagolysosomes. To exclude persistence due to apoptosis of the respective macrophages, we demonstrated viability of macrophages harboring ICL using an apoptosis assay (data not shown).

### Intracellular persistence of L-forms does not require virulence factors

To investigate whether the observed persistence of *L. monocytogenes* L-forms requires expression of virulence genes, we conducted gentamycin protection assays using attenuated, apathogenic *L. monocytogenes* knock-outs (Δ*hly*, Δ*inlA/B*, Δ*actA*, Δ*prfA*), and *L. innocua*. We found that all mutants and *L. innocua* (Figure 2) showed intracellular persistence in both P-388D1 and THP-1 macrophages, indistinguishable from wild-type L-forms (Table 2). This demonstrated that the phenomenon is independent of virulence factors. However, pre-activation of macrophages with IFN-γ prior to L-form exposure completely abrogated any intracellular persistence. Furthermore, BMM, which exhibit a stronger bactericidal activity (García-Rodas et al., 2011), also prevented persistence of intracellular L-forms. In contrast, activation of macrophages by addition of LPS or heat inactivated *L. monocytogenes* did not affect persistence of ICL. In conclusion, BMM and IFN-γ activated phagocytes do not support L-form persistence, probably based on induction of factors which are not triggered by L-forms alone (Table 2).

**Table 2:** **Challenge of various cell types with *Listeria* L-forms**.

**Cell line**	***Listeria* L-forms**	Activation^*^	**Uptake**	**“Persistence”**
P-388D1	*L. monocytogenes*-GFP	None	+	+
P-388D1	*L. innocua*-GFP	None	+	+
P-388D1	*L. monocytogenes* Δ*hly*-GFP	None	+	+
P-388D1	*L. monocytogenes* Δ*prfA*-GFP	None	+	+
P-388D1	*L. monocytogenes* Δ*AactA*-GFP	None	+	+
P-388D1	*L. monocytogenes* Δ*inlA/B*-GFP	None	+	+
P-388D1	*L. monocytogenes*-GFP	*Listeria* (h.i.)	+	+
P-388D1	*L. monocytogenes*-GFP	LPS	+	+
P-388D1	*L. monocytogenes*-GFP	IFN-y	+	−
THP-1	*L. monocytogenes*-GFP	None	+	+
THP-1	*L. monocytogenes*-GFP	IFN-y	+	−
BMM	*L. monocytogenes*-GFP	None	+	−
CACO-2	*L. monocytogenes*-GFP	None	−	−
HBMEC	*L. monocytogenes*-GFP	None	−	−
Hep-G2	*L. monocytogenes*-GFP	None	−	−

* *Activation of macrophages was performed with either 100 ng/ml IFN-y or LPS, or by addition of heat inactivated (h.i.) Listeria (MOI = 200)*.

### *Listeria* L-forms elicit an innate immune response

**Figure 5:**
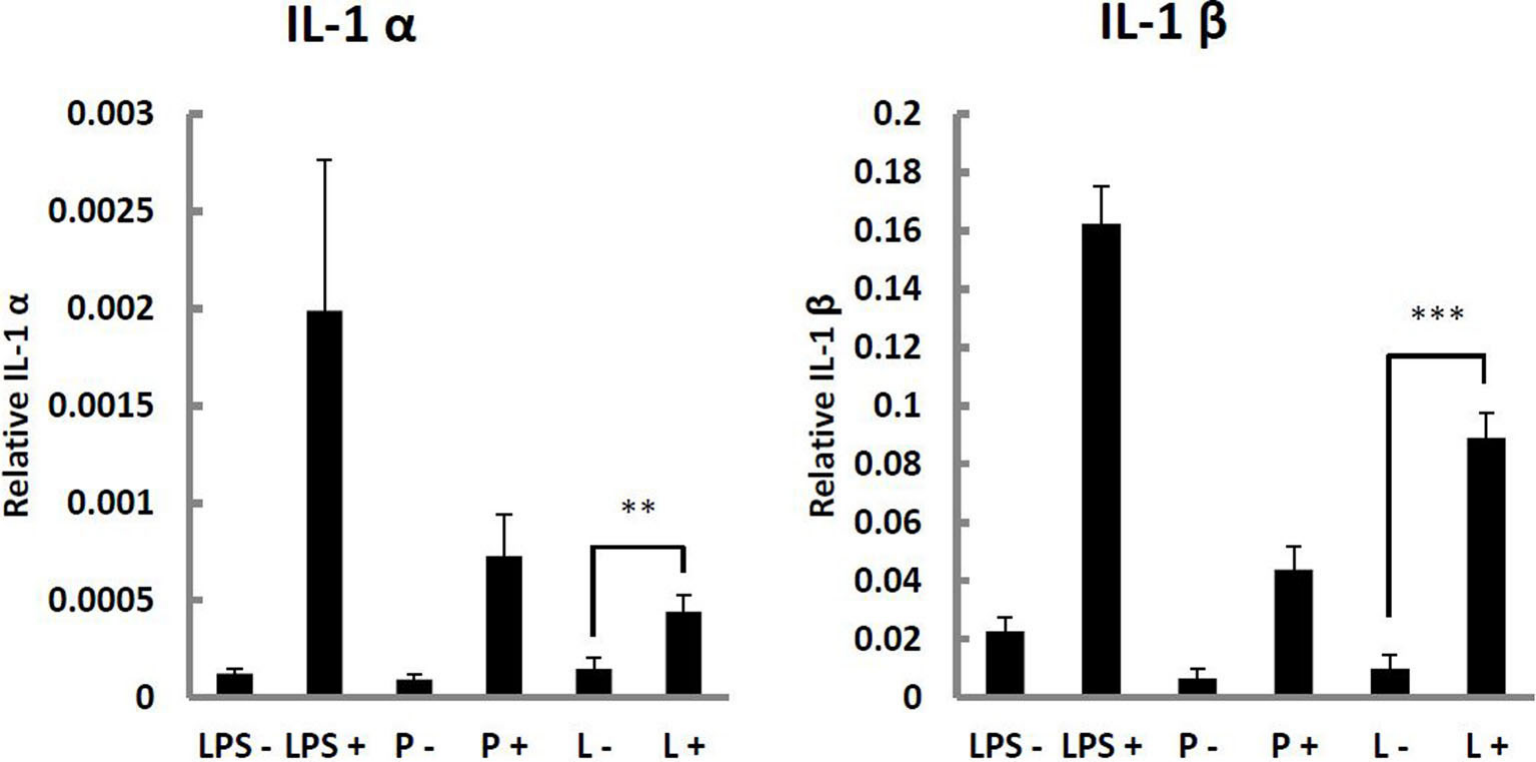
**IL-1α and IL-1β response of P-388D1 murine macrophages non-activated (−), or activated using 100 ng/ml IFN-γ (+), and challenged with *L. monocytogenes* ScottA-GFP parental (P) or L-form (L) cells, and 100 ng/ml lipopolysaccharide (LPS) as a control**. Cytokine gene expression was determined by Real-Time qRT-PCR. Values were normalized to *β-actin* levels, and values for negative controls (soft agar and medium controls) were subtracted. Data represent means ± s.e.m. Statistical analysis was performed using the unpaired Student's *t*-test: ^**^P < 0.01; ^***^P < 0.001.

Recognition of pathogens by innate immunity is based on display and recognition of specific PAMPs. Parental *L. monocytogenes* are sensed by host TLR-2, which recognizes bacterial peptidoglycan (Torres et al., 2004). As mature peptidoglycan is not present on the surface of the wall-less L-forms, we investigated whether and how they could be recognized by innate immunity. We compared the immune stimulatory potential of parental or L-form *L. monocytogenes* by quantitative analysis of pro-inflammatory cytokine mRNA levels in IFN-γ activated and non-activated macrophages. IFN-γ activated macrophages challenged with parental and L-form *L. monocytogenes* revealed a significant increase in IL-1α, and IL-1β cytokine mRNA production (Figure 5). The respective expression levels determined for parental and L-form *L. monocytogenes* were in a similar range, suggesting that the wall-less L-forms could still be recognized by activated macrophage.

### Absence of IFN-β induction and TLR-independent cytokine production indicates vacuolar localization of intracellular L-forms

**Figure 6:**
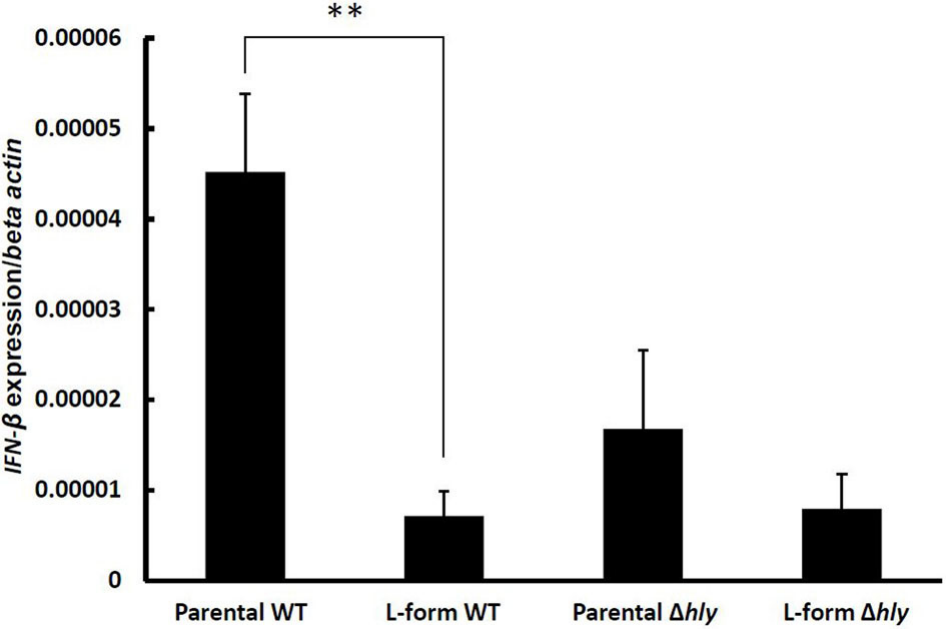
**Interferon-β (IFN-β) induction in macrophages infected by wild type, and Δ*hly* ScottA-GFP (parental and L-form)**. Gene expression was measured by Real-Time qRT-PCR, values were normalized to the house-keeping gene *β-actin*, and expression levels of negative controls (soft agar, medium) were subtracted. Data represent means ± s.e.m. Statistical analysis was performed using the unpaired Student's *t*-test: ^**^P < 0.01.

*Listeria* DacA is an adenylate cyclase responsible for generation of the type I Interferon-inducing ligand cyclic di-AMP (Woodward et al., 2010). We found a strong up-regulation of *dacA* in L-form *L. monocytogenes*. This prompted us to investigate the Interferon β (IFN-β) induction capacity of L-forms, because its presence may provide information regarding the localization of intracellular L-forms in non-activated macrophages. IFN-β mRNA levels were determined in non-activated macrophages following challenge with parental and L-form *L. monocytogenes*. As controls, Δ*hly* knockout parental bacteria and L-forms were used. A significant increase in IFN-β expression was found for wild type parental *L. monocytogenes*, which was abrogated in the Δ*hly* mutant (Figure 6). In contrast, both wild type and Δ*hly* knockout L-forms failed to induce significant levels of IFN-β (Figure 6), suggesting a vacuolar localization of intracellular L-forms.

**Figure 7:**
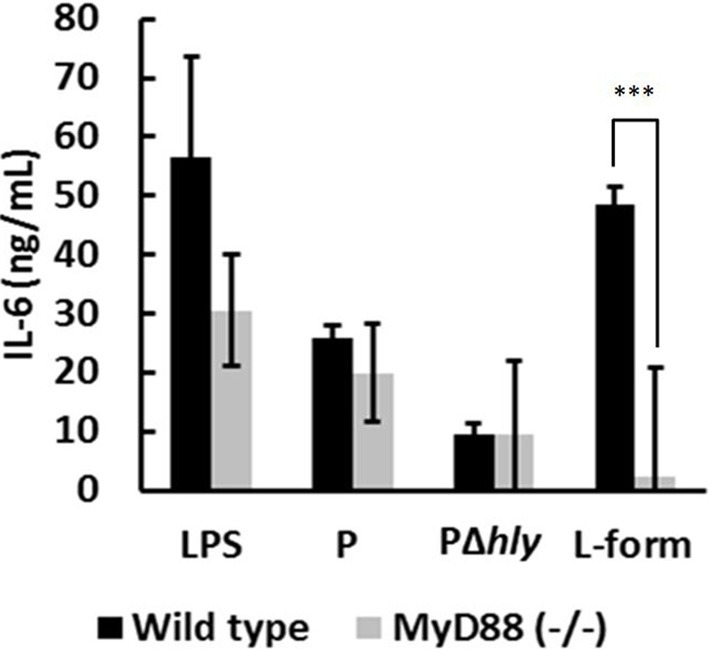
**IL-6 induction of wild type and MyD88-deficient DC challenged by LPS, parental (P), *hly*-deficient (PΔ*hly*), and L-form *L. monocytogenes***. Soft agar and medium controls were subtracted. The abrogated IL-6 response of DC challenged with L-forms indicates to an exclusively MyD88-dependent response, which suggests vacuolar localization of L-forms. Data represent means ± s.e.m. Statistical analysis was performed using the unpaired Student's *t*-test: ^***^P < 0.001.

MyD88 is an important adaptor molecule in innate immunity, which integrates TLR-derived signals for downstream activation of the NFκ B pathway (Beutler et al., 2003; Takeda and Akira, 2004). The vacuolar response to *L. monocytogenes* was reported to be entirely MyD88 dependent (Leber et al., 2008), although cross-talk to NLR-dependent pathways was described for IFN-γ activated macrophages (Herskovits et al., 2007). Thus, abrogation of IL-6 induction in MyD88-deficient DC would indicate a vacuolar localization of the bacteria. In fact, wild type but not MyD88-deficient DC produced IL-6 in response to L-form *L. monocytogenes* (Figure 7) whilst both, wild type and MyD88-deficient DC produced IL-6 in response to parental *L. monocytogenes* (Figure 7). This exclusive MyD88 dependent IL-6 response to L-forms provides further evidence for a vacuolar localization of *L. monocytogenes* L-forms, which seem not be able to escape from the phagosome into the cytosol.

### Mice challenged with *L. monocytogenes* L-forms show a strong, but transient cytokine response

**Figure 8:**
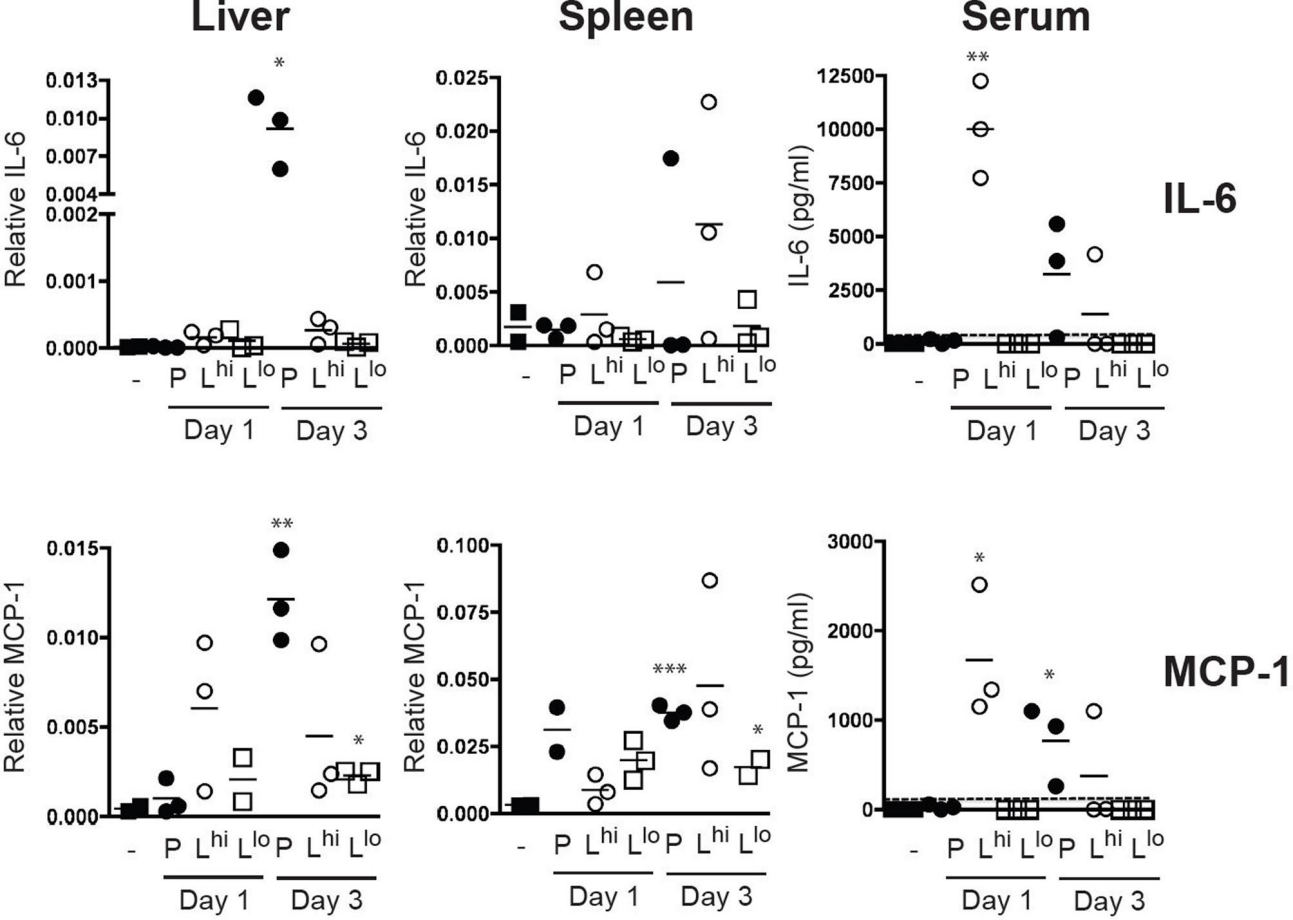
**IL-6 and MCP-1 production in BALB/c mice challenged by intraperitoneal injection of 0.5 ml protoplast buffer plus soft agar (3/1; v/v) containing 100 cfu parental (P) *L. monocytogenes*, 10^8^ cfu L-forms as high dose (L^hi^) or 10^5^ cfu L-forms as low dose (L^lo^)**. Control animals received 0.5 ml protoplast buffer plus soft agar (3/1; v/v) alone. Cytokine mRNA expression values were normalized to the GAPDH house keeping gene. Cytokine protein values are indicated in pg/ml determined by an internal standard. Dotted lines indicate the limit of detection. Statistical analysis was performed using the unpaired Student's *t*-test (two tailed, 95% confidence interval) comparing all treatment groups to the control: ^*^P < 0.05, ^**^P < 0.005, ^***^P < 0.0005.

*In vivo* pathogenicity of L-forms was tested by intraperitoneal infection of BALB/c mice with parental or L-form *L. monocytogenes*. Spleen, liver and serum of infected mice were collected at days 1, 3, and 6 p.i., to quantify IL-6 and MCP-1 (monocyte chemotactic protein). Bacterial counts in spleen and liver from mice infected with parental *L. monocytogenes* revealed that bacteria replicated *in vivo* increasing from 420 (±121) cfu on day 1, to 4570 (±2444) cfu on day 3 p.i. and were cleared after 6 days. Production of IL-6 and MCP-1 triggered by infection with parental bacteria peaked at day 3 p.i. (Figure 8). In contrast, infection with L-form cells resulted in cytokine production only for the high dose infection (10^8^ cfu), peaking at day 1, with a high variability amongst infected animals after 3 days (Figure 8). After 6 days p.i. no cytokine production was observed in animals infected with either parental or L-form *L. monocytogenes*. These data confirm our *in vitro* findings by showing that L-forms are recognized by innate immunity and can elicit strong pro-inflammatory cytokine production. Furthermore, they support the assumption that L-form cells are unable to replicate during this time period, and are eventually and effectively cleared by the host.

## Discussion

Phagocytes such as macrophages and DC are an integral part of the innate immune system, and constitute the first line of defense against pathogens (Rasmussen et al., 2009). They recognize and engulf microbial targets by phagocytosis and subject them to digestion via the phagolysosomal pathway (Flannagan et al., 2011). However, intracellular bacterial pathogens such as *Listeria monocytogenes* have developed strategies to evade degradation, such as timely escape from the phagosome (Hamon et al., 2012), which is facilitated by virulence factors expressed under control of the master regulator PrfA (De Las Heras et al., 2011). Obviously, the major question was whether *L. monocytogenes* L-forms would potentially retain their pathogenic phenotype. It has been previously shown that *L. monocytogenes* L-forms continue to express the entire set of virulence genes (Dell'Era et al., 2009; Briers et al., 2011). However, these experiments were performed at an incubation temperature of 32°C, which does not support full expression of *L. monocytogenes* virulence genes, because PrfA-dependent transcription is attenuated below 37°C (Freitag et al., 2009; De Las Heras et al., 2011). To better address this issue, we here used incubation at 37°C, and found that *hly* expression in *Listeria* L-forms is significant down-regulated compared to parental bacteria. This agrees well with the lower transcript levels of other PrfA-controlled genes such as *actA* and *hpt*. Since ActA is proposed to interfere with autophagy recognition of the bacteria in eukaryotic cells (Yoshikawa et al., 2009), its presence on the L-form surface was assessed. However, we were unable to detect ActA made by L-form cells, which again is in line with the attenuation of *actA* expression. Taken together, these findings would predict that the phagocytosed bacteria would no longer be able to escape into the cytosol and to spread into neighboring cells, and activation of the cytosolic autophagy pathway would eventually result in elimination of intracellular L-form cells (Birmingham et al., 2008; Yoshikawa et al., 2009).

Innate immune recognition of bacteria depends on the presence of PAMPs, such as peptidoglycan itself. Because this is absent in L-forms, it was interesting to see whether the defect may possibly confer a stealth character to the L-form cells, resulting in a (partial) lack of recognition by the immune system. We compared the immune stimulatory potential of parental or L-form *L. monocytogenes* in IFN-γ activated and non-activated macrophages. Challenge of IFN-γ activated P-388D1 murine macrophages with parental and L-forms revealed a significant increase in IL-1α, and IL-1β cytokine mRNA production, suggesting that the wall-less L-forms could still be recognized by activated macrophages and that absence of the mature peptidoglycan cell wall in L-forms does actually not result in an abrogated recognition by the innate immune system. This might be explained by the fact that although L-forms lack peptidoglycan-associated molecules such as Internalin A, membrane-anchored molecules such as lipoteichoic acids or lipoproteins are still present (Dell'Era et al., 2009), and they may act as PAMPs and ligands for different TLRs (Aderem and Ulevitch, 2000). L-forms also contain NLR activators such as muramyl pentapetide (including *meso*-DAP) on their surface (Dell'Era et al., 2009), which likely also represents an innate immunity target.

In non-activated macrophages, IL-1α and IL-1β cytokine production was not induced by L-forms. This is in line with the observed persistence of viable *Listeria* L-forms in non-activated macrophages, which was independent from the virulence traits of the bacteria. In contrast, persistence was never observed for IFN-γ activated phagocytes. Moreover, BMM very effectively eradicated L-form *L. monocytogenes*. Interestingly, persistence in macrophages has also been reported for *S. aureus* L-forms. After intra-phagosomal formation of *S. aureus* L-forms in alveolar macrophages of rats, the phagocytes were incapable of lysosomal acid-phosphatase activation and phagosome-lysosome fusion (Michailova et al., 2007), which could suggest that these L-forms may represent persisters (Beaman and Scates, 1981; Domingue and Woody, 1997). Consequently, the wall-less state of bacteria had been speculated to possibly assume roles in chronic disease, based on the lack of immune recognition (Domingue and Woody, 1997; Domingue, 2010). However, this hypothesis is clearly not supported by our findings. Strong induction of cytokine response following exposure to L-form *L. monocytogenes in vitro* and *in vivo* demonstrates that the wall-less cells are still recognized by the host, and do elicit a pro-inflammatory cytokine production, at least in activated immune cells.

IFN-β is exclusively induced after cytosolic entry of *L. monocytogenes*, and not by escape-deficient mutants (O'Riordan et al., 2002; Stockinger et al., 2002). Comparing IFN-β induction of parental and L-form *L. monocytogenes* with their Δ*hly* mutants revealed that only parental *L. monocytogenes* can induce significant levels of IFN-β, whereas L-forms behave similar to Δ*hly* mutants. This observation indicates that L-forms are unable to escape into the cytosol, but remain trapped in the phagosome. Further evidence for this assumption was provided by infecting MyD88-deficient DC. The immune response from vacuolar *Listeria* was shown to be completely dependent on MyD88, and no role for other adaptor molecules was found (Leber et al., 2008). Investigation of the IL-6 response by MyD88-deficient DC challenged with parental and L-form *L. monocytogenes* revealed an abrogated IL-6 induction by L-forms in MyD88-deficient DC, in contrast to parental *L. monocytogenes*. This exclusive MyD88-dependent signaling induced by L-forms indicates a phagosomal localization of intracellular L-forms in macrophages. The short-lived response following *in vivo* challenge with viable L-forms shows that these cells are unable to escape from the phagosome, and instead are cleared via the phagolysosomal pathway. Although our data are preliminary and need confirmation, they still provide sufficient support to assume that *Listeria* L-forms are not able to escape from phagosomes.

In conclusion, our study demonstrated attenuation of pathogenicity in stabilized *L. monocytogenes* L-form cells lacking a cell wall. Interaction with innate immune cells, and the host response to L-forms revealed they were still recognized by the innate immune system. Lack of IFN-β induction and the exclusive MyD88-dependent signaling both suggest a phagosomal localization of intracellular L-forms in macrophages, which correlates with the lack of *hly* expression. However, the reasons for the low-frequency persistence of L-forms in non-activated macrophages remain unclear.

Future research should also be directed toward the possible role of transient L-form cells, which feature an only partial loss of the wall, and can revert to walled cells, in infections or chronic disease. In fact, such transient L-forms are much more likely to occur under normal environmental circumstances, where the bacteria may encounter conditions that interfere with cell wall synthesis, such as improper use of beta-lactam antibiotics, or lytic enzymes produced by phage or bacterial competitors. Paradoxically, use of antibiotics to treat infections could contribute to the generation of L-forms from pathogenic bacteria. Because of their potential to revert to the parental state, transient L-forms may exhibit a more significant role in bacterial infections.

### Conflict of interest statement

The authors declare that the research was conducted in the absence of any commercial or financial relationships that could be construed as a potential conflict of interest.
