# Supplementary material for: Cell-wall deficient L. monocytogenes L-forms feature abrogated pathogenicity
Source: Front Cell Infect Microbiol. 2014 May 20;4:60. doi: 10.3389/fcimb.2014.00060 (PMC4033035; doi:10.3389/fcimb.2014.00060)
Supplement: Supplementary file 1 [file DataSheet1.PDF]

Supplementary Material

Table S1: Oligonucleotide primers used in this study

Primer name	Sequence (5'→3')	T_m (°C)
**Construction of a *hly* deletion mutant**		
*hly*_f_del_A	ATC A**GA GCT C**GT TGT GTC AGG TAG AGC G	63.8
*hly*_r_del_B	GAT AAA GTG TAG TGC CAT CCT TTG CTT CAG TTT G	65.2
*hly*_f_del_C	GAA GCA AAG GAT GGC ACC ACG CTT TAT CCG	64.4
*hly*_r_del_D	AAA A**GG ATC C**TT GTA TAT TAT TTC GGA GCC TTT CTT C	65.4
hly-x-FWD	CTT ATA CAA AAT GGC CCC CTC C	59.9
hly-y-REV1	TTC ATC CGC GTG TTT CTT TTC G	61.4
hly-z-REV2	ATT TTT CGT GTG TGT TAA GCG G	59.5
**Quantification of gene expression by Real-Time qRT-PCR**		
actA FWD	AAG TGG CGA AAG AGT CAG TTG C	62.7
actA REV	ACT TTT AGG GAA AAA TGG TTG TTG GT	60.3
gyrA FWD	GCC GTG GTT CGA TTA CTG TT	55.4
gyrA REV	GTG ATG CCG TCG ATT TTC TT	53.4
hly FWD	ATA GCA CCA CCA GCA TCT CC	60.1
hly REV	GGC ACA TTT GTC ACT GCA TC	60.1
dacA FWD	CGT TTC CAC ATC TTG CAT TG	60.1
dacA REV	TTG CGT TAC GGG TTT TAT CC	59.8
pgdA FWD	CAC CGG AAC CGA AGA AAG TA	60.1
pgdA REV	CGA GGA ACT TGG ACC ATC AT	59.9
lgt FWD	CAC CAA TTA GCG CAC CAT AA	59.6
lgt REV	GAT TTG CTG ATT TGG GCA AT	59.9
hpt FWD	AGG GTA TGT CGT GGA TGG AA	59.6
hpt REV	CAT TTG CTC CCC AAA GGA TA	59.9
iap FWD	TGG TGC AGC TTG TTT TTC AG	60.0
iap REV	ACG GTG TTT CCG TTC AAG AC	60.0
beta actin FWD	CCC TGA AGT ACC CCA TTG AAC	60.6
beta actin REV	CTT TTC ACG GTT GGC CTT AG	59.7
IFN β FWD	CAC AGC CCT CTC CAT CAA CT	60.2
IFN β REV	AGT CTC ATT CCA CCC AGT GC	60.1
IL-1 α FWD	CAC CTT ACA CCT ACC AGA GTG ATT TG	62.2
IL-1 α REV	TGT TGC AGG TCA TTT AAC CAA GTG	63.3
IL-1 β FWD	CAG GAT GAG GAC ATG AGC ACC	63.0
IL-1 β REV	GGA ACG TCA CAC ACC AGC AG	62.8

Restriction sites given in bold letters
